# Chloroplast sequence variation and the efficacy of peptide nucleic acids for blocking host amplification in plant microbiome studies

**DOI:** 10.1186/s40168-018-0534-0

**Published:** 2018-08-18

**Authors:** Connor R. Fitzpatrick, Patricia Lu-Irving, Julia Copeland, David S. Guttman, Pauline W. Wang, David A. Baltrus, Katrina M. Dlugosch, Marc T. J. Johnson

**Affiliations:** 1 Department of Ecology & Evolutionary Biology, University of Toronto, Toronto, ON M5S 3B2 Canada; 2 Department of Biology, University of Toronto Mississauga, Mississauga, ON L5L 1C6 Canada; 3 Department of Ecology & Evolutionary Biology, University of Arizona, Tucson, AZ 85721 USA; 4 Centre for the Analysis of Genome Evolution & Function, University of Toronto, Toronto, ON M5S 3B2 Canada; 5 Department of Cell & Systems Biology, University of Toronto, Toronto, ON M5S 3B2 Canada; 6 School of Plant Sciences, University of Arizona, Tucson, AZ 85721 USA

**Keywords:** Plastid variation, PNA clamp, Host contamination, 16S amplicon sequencing, Asteraceae

## Abstract

**Background:**

The ability to efficiently characterize microbial communities from host individuals can be limited by co-amplification of host organellar sequences (mitochondrial and/or plastid), which share a common ancestor and thus sequence similarity with extant bacterial lineages. One promising approach is the use of sequence-specific peptide nucleic acid (PNA) clamps, which bind to, and block amplification of, host-derived DNA. Universal PNA clamps have been proposed to block host plant-derived mitochondrial (mPNA) and plastid (pPNA) sequences at the V4 16S rRNA locus, but their efficacy across a wide range of host plant species has not been experimentally tested.

**Results:**

Using the universal PNA clamps, we amplified and sequenced root microbial communities from replicate individuals of 32 plant species with a most recent common ancestor inferred at 140 MYA. We found the average rate of host plastid contamination across plant species was 23%, however, particular lineages exhibited much higher rates (62–94%), with the highest levels of contamination occurring in the Asteraceae. We investigated chloroplast sequence variation at the V4 locus across 500 land plant species (Embryophyta) and found six lineages with mismatches between plastid and the universal pPNA sequence, including all species within the Asteraceae. Using a modified pPNA for the Asteraceae sequence, we found (1) host contamination in Asteraceae species was reduced from 65 to 23%; and (2) host contamination in non-Asteraceae species was increased from 12 to 69%. These results demonstrate that even single nucleotide mismatches can lead to drastic reductions in pPNA efficacy in blocking host amplification. Importantly, we found that pPNA type (universal or modified) had no effect on the detection of individual bacterial taxa, or estimates of within and between sample bacterial diversity, suggesting that our modification did not introduce bias against particular bacterial lineages.

**Conclusions:**

When high similarity exists between host organellar DNA and PCR target sequences, PNA clamps are an important molecular tool to reduce host contamination during amplification. Here, we provide a validated framework to modify universal PNA clamps to accommodate host variation in organellar sequences.

**Electronic supplementary material:**

The online version of this article (10.1186/s40168-018-0534-0) contains supplementary material, which is available to authorized users.

## Background

Advances in molecular tools have led to unprecedented insight into host-associated microbiota and their importance for host nutrition, development, and immunity [1–4]. Microbial assemblages once observable only through culture-dependent or low-throughput approaches are now being more completely characterized via high-throughput approaches, revealing tremendous previously unobserved diversity [5, 6]. Efficient next generation sequencing methods enables researchers to describe variation in microbial diversity across hosts, environments, and experimental designs, leading to greater understanding of their role in the ecology and evolution of host organisms.

Host-associated microbial communities are frequently characterized using deep amplicon sequencing [7], with bacterial taxa typically identified by their sequences at one or more of the hypervariable regions of the 16S rRNA gene [8]. Amplification of these regions from host tissue can result in reduced sequencing efficiency due to host contamination, which is caused by sequence similarity due to the common ancestry shared between eukaryotic organelles and bacteria [9]. For example, when sequencing the V4 region of the 16S rRNA gene from plant tissue, host-derived plastid and mitochondrial sequences can account for as much as 95% of all sequenced reads from a given sample [10]. Such high-host contamination limits the number of samples that can be sequenced simultaneously with sufficient read depth, and quickly erodes the value of next generation sequencing approaches in describing the microbiome. Consequently, techniques to limit host contamination are imperative to studies of host-associated microbiota.

A recent approach that circumvents host contamination during PCR is the use of peptide nucleic acid (PNA) oligos, which block the amplification of host DNA [10]. PNA oligos are artificially synthesized polymers of pyrimidine and purine bases linked together via peptide bonds [11], which obey typical Watson-Crick base pairing and form thermally stable hybrid complexes with DNA and RNA [12, 13]. These unique properties have led to a long history of PNA use in biotechnology and medical research [13–16]. Due to their sequence specificity, PNAs can be designed to bind host organellar sequence variants of a target region and effectively block their amplification during PCR. Recently, Lundberg et al. [10] pioneered this approach in plant microbiome studies, designing universal PNA oligos to block the amplification of plant plastid (pPNA) and mitochondrial (mPNA) 16S V4 sequences. The sequences of the pPNA and mPNA were selected, in silico, for high complementarity to organellar-derived V4 sequences across land plant species (reference [10]: Supplementary Figure 13), and low complementarity to V4 sequences derived from a 16S rRNA database of extant bacterial lineages (reference [17]: Greengenes database). However, the efficacy of these universal PNAs was tested only in the model plant *Arabidopsis thaliana.* Thus, there is a need for a broader evaluation of universal PNAs across a phylogenetically diverse sample of plant species before the universal nature of these sequences can be firmly established.

We used the universal PNA clamps designed by Lundberg et al. [10] in a characterization of the root microbiomes of 32 flowering plant species from 14 families, with a most recent common ancestor inferred at 140 MYA. We investigated how rates of plastid contamination vary across host plant species, and whether these are associated with mismatches between universal pPNA and target host organellar sequences. We then evaluated if plastid contamination could be reduced through simple modification to correct sequence mismatches, and whether such modification results in altered diversity estimates within or between bacterial communities. Our study is the first to test the efficacy of widely-used universal PNAs across host diversity and to evaluate the utility of developing custom PNA sequences for the study of microbiota associated with individual host species.

## Methods

### Sample collection

Of the 32 plant species sampled in this study, 30 were grown from sterilized seed in a common environment in the summer of 2014, and two were sampled in the field (*Centaurea solstitialis, Rhexia virginica*; Additional file 1: Table S1). In the common environment, we used seeds collected between 1999 and 2014 from numerous plants in single open-pollinated populations across southern Ontario, which were stored frozen at − 20 °C and surface sterilized using ethanol and bleach prior to germination on 1% agar media plates with half-strength MS nutrients. Seedlings were transplanted into 1 L pots containing a combination of live inoculum collected from the University of Toronto’s field station (Koffler Scientific Reserve) and sterilized soil, and then grown in a growth chamber for 2 weeks before transport to a roof-top common garden at the University of Toronto Mississauga. After 12 weeks of growth, we collected root endosphere samples from every individual using an established protocol described by Fitzpatrick et al. [18].

The *C. solstitialis* root endosphere sample was collected from three plants across three populations in France, Spain, and the USA in June 2015, using protocols described by Lu-Irving et al. [19]. We collected *R. virginica* individuals from natural populations occurring along the western shore of Long Lake, Ontario in August 2017. We used a trowel to remove an individual plant and surrounding soil core from three discrete plant patches separated by at least 100 m. These samples were processed identically to rooftop-grown samples within 3 h of collecting.

We extracted total DNA from root endosphere samples using the MoBio Powersoil DNA (QIAGEN, 12955–4) extraction kit following the manufacturer’s protocol. Prior to DNA extraction, root tissue was ground either manually in liquid nitrogen (*C. solstitialis*) or in a liquid nitrogen cooled tissue homogenizer (all other species, Retsch CryoMill 20.749.0001).

### Library preparation and sequencing

We amplified the V4 region of the 16S rRNA gene from each sample using the 515F and 808R primers with dual-indexed barcodes under standard PCR conditions [20], for 24 cycles (the experimentally determined minimum number of cycles required for optimal amplification of root endosphere samples). Reactions were performed in triplicate in 96-well plates, each of which included negative, positive, and mock community controls. Following amplification, triplicate reactions were combined and quantified, samples were pooled in equimolar concentrations, and the final library was purified and quantified. Sequencing was performed on an Illumina MiSeq (Illumina) using 2 × 150 bp paired-end reads. See Additional file 1: Supplemental methods for a detailed description of PCR conditions.

**Fig. 1:**
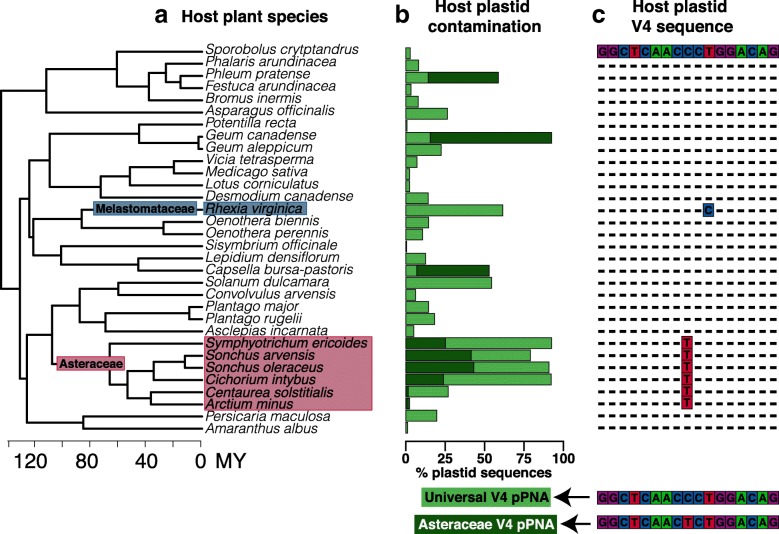
Plastid contamination across host plant species is driven by variation in host plastid V4 sequence. **a** Root microbiota were characterized from 32 angiosperm species using universal pPNA during PCR. **b** Plastid contamination with universal pPNA (light green bars) significantly varied across species (Likelihood ratio test *χ*^2^ = 108, *p* < 0.001), but was consistently elevated in the Asteraceae (red shading). Note that bars are not stacked for host species amplified with both universal and modified pPNA. Each bar depicts the mean contamination rate observed with the respective pPNAs (e.g. *Symphyotrichum ericoides* exhibits a mean plastid contamination of 25% and 94% with the universal and modified pPNAs, respectively). **c** Members of the Asteraceae exhibit a single nucleotide mismatch compared to the universal pPNA sequence (shown below the multiple sequence alignment Original V4 pPNA). Amplified with a modified pPNA (shown below the multiple sequence alignment Asteraceae V4 pPNA), plastid contamination was reduced in Asteraceae host species (ANOVA *F*_5,1_ = 31.07, *p* = 0.003), and elevated in non-Asteraceae host species (dark green bars ANOVA *F*_2,1_ = 23.42, *p* = 0.04). We further tested whether single nucleotide mismatches in chloroplast V4 sequence leads to elevated plastid contamination rates by characterizing the root microbiome of *Rhexia virginica* (blue shading). This species belongs to the Melastomataceae, a lineage predicted to exhibit elevated plastid contamination due to an independent nucleotide substitution in chloroplast V4 sequence (see Fig. 2)

Each PCR included 1 μM mPNA and pPNA clamps. By examining chloroplast sequences of Asteraceae species available in NCBI GenBank and obtained by the authors (K. Dlugosch, unpub.; Additional file 2: Table S3), a single nucleotide mismatch was identified between the universal pPNA sequence (5’-GGCTCAACCCTGGACAG-3′) and its target region in Asteraceae, and a modified pPNA clamp was designed to match the Asteraceae chloroplast sequence (5’-GGCTCAACTCTGGACAG-3′). Reactions including universal (original) pPNA clamps were performed on all samples (32 species). Reactions that included the modified clamp were performed on each of the Asteraceae samples (six species) and three representative non-Asteraceae species (Fig. 1; Additional file 1: Table S2). All reactions included universal mPNA clamps (5’-GGCAAGTGTTCTTCGGA-3′).

### Microbiome bioinformatics

After trimming primer and index sequences and demultiplexing, we processed sequencing reads using the R package “DADA2” [21]. We trimmed 5 bp from the start and 10 bp from the end of each paired end sequence due to poor *Q* scores, and filtered sequences with any instance of a *Q* score less than 2, an ambiguous nucleotide assignment, or with greater than 2 expected errors per read.

Using DADA2, we inferred amplicon sequence variants (ASVs). To parameterize an error model for each sequencing run we used either 50 random samples (each with > 75,000 reads), or all samples if the sequencing run included fewer than 50 samples. This procedure controlled for differences in sequencing performance across runs. After identifying ASVs, we merged forward and reverse sequences and removed chimeras, for a total of 57,116 ASVs.

We used the Ribosomal Database Project naïve Bayesian classifier [22] and the SILVA reference database v.128 [23] to assign taxonomy to individual ASVs. Using PASTA, we aligned ASV sequences and built a maximum likelihood phylogenetic tree [24]. Next, we used the R package “phyloseq” to further process our samples [25]. Specifically, we removed ASVs that lacked a kingdom assignment or were assigned to Archaea or Eukaryota (3597 ASVs), or that lacked a phylum assignment (3521 ASVs). We also removed ASVs classified as mitochondria and individual samples with fewer than 800 sequences. ASVs classified as plastid were retained for our analysis of plastid contamination, but removed for our analysis of the effects of pPNA type on bacterial diversity.

For diversity analyses, we applied a prevalence and abundance cut-off where ASVs had to be found in at least two samples at an abundance of at least 25 sequence reads per sample. This yielded 1470 ASVs, which accounted for 96% of the total number of sequences in this entire dataset. We calculated *α*-diversity as observed species richness (*R*), inverse Simpson’s diversity (*D*^−1^), and evenness (calculated as *D*^−1^/*R*) [26]. We then performed proportional abundance normalization on this common set of ASVs, where the sequencing reads for an ASV in a given sample were divided by the total number of sequencing reads in that sample [27].

### Statistical analyses

#### Effect of pPNA type on plastid contamination

We used linear mixed models (LMMs; function “lmer” from the R package “lme4” [28]) to analyze levels of plastid contamination among samples amplified using either universal or modified pPNAs. Using the “logit” function from the R package “car” [29], we quantified plastid contamination as the logit transformed proportion of sequenced reads classified as plastid for each sample [30]. First, we tested whether plant species varied in their rates of plastid contamination when root endosphere DNA was amplified using V4 primers and the universal pPNA. We used LMMs with the number of usable reads as a fixed covariate and plant species as a random effect. Next, we tested whether plant species from the Asteraceae exhibited reduced plastid contamination when root endosphere DNA was amplified with a modified Asteraceae-pPNA sequence. For this analysis pPNA type and the number of usable reads were coded as fixed effects, and plant species and the interaction between pPNA type and plant species were coded as random effects. Finally, we tested whether non-Asteraceae plant species exhibited increased contamination when root endosphere DNA was amplified with the Asteraceae-pPNA. Here, pPNA type and the number of usable reads were coded as fixed effects and plant species and the interaction between pPNA type and plant species were coded as random effects. Using the model object from “lmer,”, we tested the significance of fixed effects with type III ANOVA from the R package “car” using the Kenward-Roger degrees of freedom approximation (Additional file 1: Table S4). To test the significance of random effects we used function “rand” from the package “lmerTest” [31] to perform likelihood ratio tests comparing full and reduced models (Additional file 1: Table S4).

#### pPNA target site variation across land plants

Whole chloroplast genome sequences from land plants (Embryophyta) were downloaded from GenBank (589 sequences). When there were sequences for multiple species in the same genus, only the longest sequence was retained. This yielded a set of chloroplast genomes for 500 land plant genera (Additional file 2: Table S3). The 16S V4 region for each genome in the set was identified using BLAST, and aligned using MAFFT [32]. The alignment was visually inspected at the universal pPNA clamp target site to identify genera with mismatches to the clamp sequence.

#### Effect of pPNA type on the amplification of individual bacterial taxa

We used the raw read counts from the common set of ASVs agglomerated at each bacterial taxonomic rank to test whether pPNA type affected the amplification of individual phyla, classes, orders, families, genera, and ASVs. This analysis included the six Asteraceae host plant species and the three representative non-Asteraceae host plant species. To comprehensively test for differential abundance, we used the R packages “DESeq2” and “ALDEx2” (Additional file 1: Table S5), both of which were originally designed for RNA-seq data but are effective methods to test for differential abundance in microbiome studies [33]. DESeq2 fits negative binomial generalized linear models to variance stabilized count data (e.g. genes, ASVs etc.) and estimates the log_2_-fold change in abundance across experimental factors [34]. We used log-likelihood ratio tests implemented in DESeq2 to test the significance of pPNA type on the abundance of bacterial taxa. ALDex2 accounts for the compositional nature of sequencing datasets by performing a scale-invariant transformation on read counts, which are modeled as distributions of posterior probabilities sampled from a Dirichlet distribution [35, 36]. For all ALDex2 analyses, we used 128 Monte Carlo instances and the geometric mean of all taxa to perform the scale-invariant transformation. We tested the significance of pPNA type on the abundance of bacterial taxa through Welch’s *t*-tests, implemented in ALDEx2.

#### Effect of pPNA type on α-diversity and β-diversity

Using the normalized dataset of common ASVs we performed Principal Coordinates Analysis (PCoA) using a weighted UniFrac dissimilarity matrix, calculating β-diversity as the sample scores along the first three PCoA axes. We repeated the above analysis using a dataset rarefied to 800 reads per sample instead of applying a prevalence and abundance cut-off. Analyses using either dataset yielded near identical results (Additional file 1: Table S6 and Table S7), therefore we present the non-rarefied results. We used LMMs to analyze the effects of pPNA type (universal versus modified), plant species, and their interaction on microbial α-diversity and β-diversity (Additional file 1: Table S6 and Table S7). Data for α-diversity indices were ln-transformed to meet assumptions of normality and homogeneity of variance. pPNA type and usable sequences were treated as fixed effects, and plant species and the interaction between plant species and pPNA type were treated as random effects. We performed significance testing of fixed and random effects in our LMMs as outlined above. In addition to LMMs, we performed non-parametric multivariate analyses of variance (PERMANOVA using the adonis function from the “vegan” package in R [37]). We note that our sequencing strategy yielded comparable sequence read depth for root microbiota amplified with either universal or modified pPNA (universal 44,517 ± 2456; modified 40,838 ± 6845; mean ± SE). Consequently, our analyses test whether bias introduced by pPNA type and not simply altered read depth affects sample diversity. R script for statistical analyses can be found in Additional file 3.

## Results

### pPNA efficacy and chloroplast V4 sequence variation

Across host plant species the average proportion of sequenced reads classified as plastid was 20% (Fig. 1; median = 4%; standard deviation = 30%). Plastid contamination significantly varied across host plant species (0–94%; Additional file 1: Table S2; *χ*^2^ = 108, *p* < 0.001), however, particular plant clades exhibited greatly elevated contamination rates including the Asteraceae (Asteraceae 65%, non-Asteraceae 12%; Mann-Whitney-Wilcoxon test *U* = 131, *p* = 0.004), and *Rhexia virginica* from the Melastomataceae (Melastomataceae 62%, non-Melastomataceae 12%; Mann-Whitney-Wilcoxon test *U* = 10, *p* = 0.01).

**Fig. 2:**
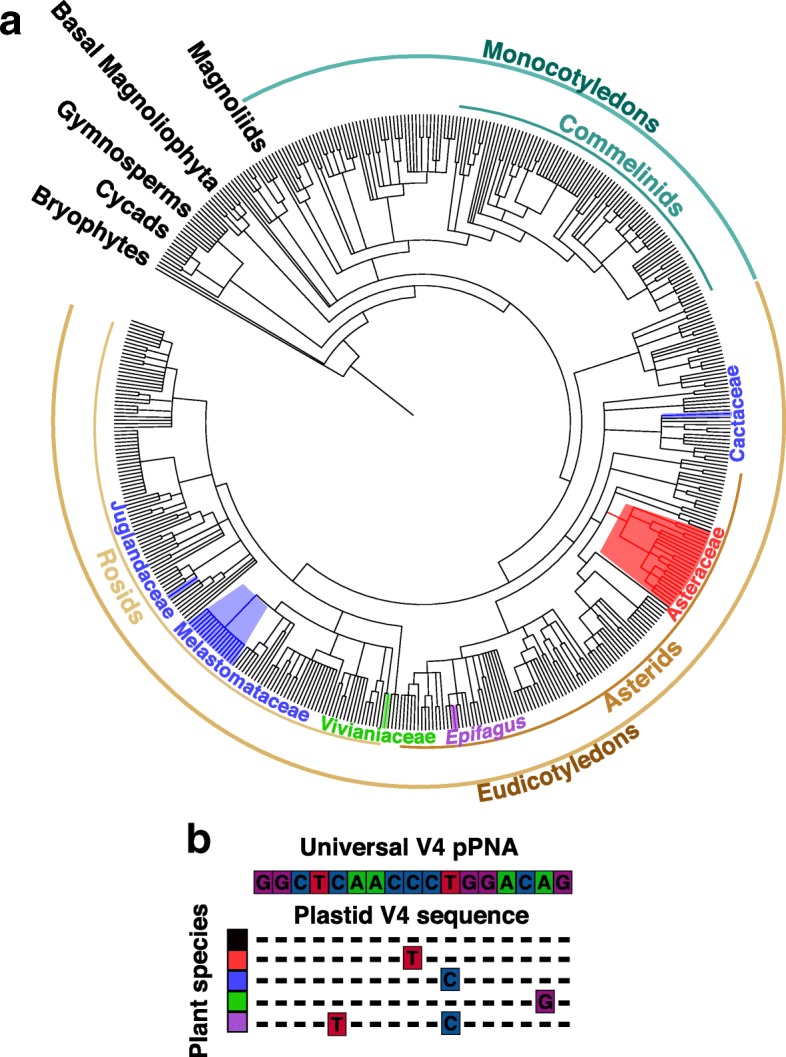
Occurrence of sequence mismatch between the universal V4 pPNA and the plastid 16S V4 locus across 500 land plant species. **a** We queried NCBI GenBank accessions and found six independent occurrences of mismatch including five plant families (Juglandaceae, Melastomataceae, Vivianaceae, Asteraceae, and Cactaceae), and one genus from the Orobanchaceae (*Epifagus*). **b** Plant taxa of the phylogenetic tree in **a** are colored according to the particular nucleotide mismatch between the universal V4 pPNA and plastid 16S V4 locus. We note that Juglandaceae, Vivianaceae, and Cactaceae are represented with only a single taxon and whether or not the observed mismatch extends to other members of these families is unknown

We investigated whether variation in chloroplast V4 sequence could be driving the elevated plastid contamination found in particular host plant species. After aligning chloroplast sequences from 500 plant species representing major land plant lineages (Additional file 2: Table S3, taxa with mismatch are shaded red), we identified six lineages, including the Asteraceae and Melastomataceae, with mismatches between plastid V4 and universal pPNA sequences (Fig. 2).

### The effects of pPNA modification on plastid contamination, the amplification of bacterial taxa, and estimates of diversity

**Fig. 3:**
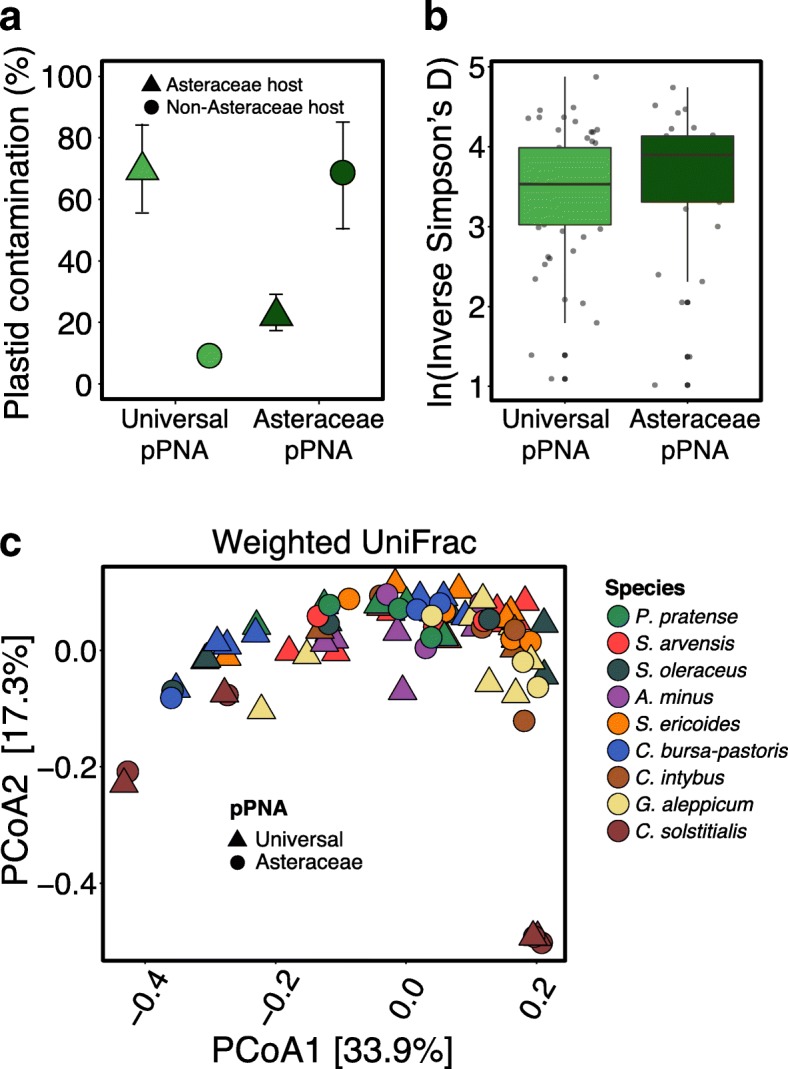
Modification to pPNA sequence reduces contamination without altering estimates of diversity. **a** Asteraceae host plant species (6 species) exhibited a reduction in contamination when amplified with modified pPNAs to reflect the single nucleotide substitution in their chloroplast V4 sequence (*F*_5,1_ = 31.07, *p* = 0.003). In contrast, non-Asteraceae species (3 species) exhibited an increase in contamination when amplified with a modified pPNA representing a single nucleotide mismatch (ANOVA *F*_2,1_ = 23.42, *p* = 0.04). **b** pPNA type had no effect on estimated diversity within (ANOVA *F*_7,1_ = 1.29, *p* = 0.29) or **c** between root microbiomes (PERMANOVA pseudo*-F* = 1.70, *p* = 0.10). Note that with the exception of *C. solstitialis,* Fig. 3 includes different DNA samples amplified with either universal or Asteraceae-modified pPNA for each host plant species. See Additional file 1: Figure S1 for an ordination of the same DNA samples amplified with both pPNAs

We observed a drastic reduction in plastid contamination across Asteraceae hosts when root DNA was amplified using our Asteraceae-modified pPNA, from 65 to 23% (Fig. 3a; Additional file 1: Table S4; *F*_5,1_ = 31.07, *p* = 0.003). When we amplified root DNA from three non-Asteraceae host plant species we observed a drastic increase in contamination, from 12 to 69% (Fig. 3a; Additional file 1: Table S4; *F*_2,1_ = 23.42, *p* = 0.04). The modified pPNA had no effect on the abundance of any bacteria taxa across all taxonomic ranks when analyzed with either DESeq2 or ALDEx2 (Additional file 1: Table S5). Importanly, across all taxonomic ranks, both methods detected very similar sets of bacterial taxa that were differentially abundant across host plant species, indicating strong agreement between both analyses (Additional file 1: Table S5). Finally, we found that the modified pPNA had no effect on the estimated bacterial diversity within root microbiomes regardless of the diversity metric (Fig. 3b; Additional file 1: Table S6; *F*_8,1_ = 1.29, *p* = 0.29). We also found little evidence that pPNA modification effects the composition of characterized root microbiomes (Fig. 3c; Additional file 1: Table S7; PERMANOVA: *R*^*2*^ = 0.02, *p* = 0.10).

## Discussion

We found significant variation across angiosperm host plant species in plastid contamination while using existing universal pPNAs (Fig. 1). Occurrences of mismatch between chloroplast V4 and universal pPNA sequence were associated with high rates of plastid contamination (Fig. 2). Modification of pPNA sequences to correct this mismatch significantly reduced plastid contamination rate without altering the amplification of individual bacterial taxa or estimates of α-diversity or β-diversity (Fig. 3). Below, we discuss the importance of these results for the design of host microbiome studies.

The ability of PNAs to block DNA amplification relies on the increased thermal stability (i.e. increased melting temperature, *T*_m_) of PNA-DNA versus DNA-DNA duplexes. Therefore, the efficacy of a mismatched PNA should depend on the reduction of *T*_m_ as a result of the mismatch [14]. Past work demonstrates that a single nucleotide mismatch between PNA and DNA sequences can result in a reduction of *T*_m_ by 8–20 °C [14, 38]. The largest Δ*T*_m_ are predicted when mismatches occur within 3–5 nucleotides of either end of the PNA molecule and result in A:A or G:G complementary bases [38]. The mismatches observed in Asteraceae (Fig. 1c; nine nucleotides from closest PNA end, resulting in C:A) and Melastomataceae (Fig. 1c; seven nucleotides from closest PNA end, resulting in T:G) would therefore not be expected to result in a large Δ*T*_m_. However, given the significant increases in host contamination, the Δ*T*_m_ caused by these mismatches must have been sufficiently large to inhibit pPNA annealing during PCR. These results emphasize the importance of host organelle sequence variation in determining the efficacy of universal PNA.

While mismatch between host plastid and pPNA sequence caused elevated plastid contamination, we also observed variation in plastid contamination across host plant species regardless of pPNA mismatch (Fig. 1b). For example, *Solanum dulcamara*, for which no evidence of sequence mismatch was found, exhibited higher average plastid contamination with universal pPNAs than other species that similarly lacked any detectable host-pPNA mismatch (*S. dulcamara* 41%, others 9%). Conversely, *Arctium minus* (Asteraceae) exhibited markedly lower contamination than most other Asteraceae species, in both the presence and absence of a pPNA mismatch. We propose three non-exclusive mechanisms that could contribute to variation in plastid contamination across host plant species independently of a pPNA mismatch. First, DNA secondary structure can inhibit PNA-DNA binding [39, 40], so structural variation in target flanking regions may result in variable efficacy of pPNA binding across a range of hosts. Second, variation in the number of plastid genomes per cell across plant species could also contribute to observed variation in plastid contamination. Third, host-specific variation in root bacterial load or bacterial DNA extraction efficiency could contribute to variation in plastid contamination. We found no evidence that average DNA extraction yield across species was related to rates of plastid contamination (*F*_1,8_ = 1.43, *p* = 0.27), indicating that extraction efficiency in general is unrelated to plastid contamination rates.

In our dataset, relative levels of chloroplast contamination from host plant sequences remained constant within species, regardless of which pPNA was used. That is, species exhibiting the highest (or lowest) contamination with universal pPNAs typically also exhibited the highest (or lowest) contamination with modified pPNAs (Additional file 1: Figure S2; Asteraceae species *R*^*2*^ = 0.58, *P* = 0.05, *n* = 6; non-Asteraceae species *R*^*2*^ = 0.86, *p* = 0.17, *n* = 3). This observation is consistent with each of the three mechanisms outlined above. Since this study focused on the effects of PNA sequence modification on host contamination, we refrain from making specific recommendations regarding non-sequence-based variation in contamination levels. However, we note that Lundberg et al. [10] demonstrated a dosage effect of pPNA concentration on host contamination, suggesting that near complete blocking of host organellar amplification may require optimization of PNA concentration by sample to accommodate potential variation in host-specific organelle genome secondary structure or copy numbers, microbial load, or microbial DNA extraction efficiency.

Across land plants, the binding site of the universal pPNA designed by Lundberg et al. [10] is highly conserved; however, our survey of 500 land plant genera identified six lineages in which there is a mismatch between the universal pPNA sequence and plastid DNA (Fig. 2). These lineages include the Asteraceae, Cactaceae, Melastomataceae, Juglandaceae, Vivianiaceae, and the genus *Epifagus* within the Orobanchaceae. Our survey consisted of sequences from whole chloroplast genomes available on NCBI GenBank, and thus may not fully represent the breadth of land plant diversity. Further study is likely to reveal other plant lineages with variant pPNA binding sites. As an obligate parasite, *Epifagus virginiana* is notable because its target site sequence contains two substitutions, which likely reflects the accelerated substitution rate of chloroplast sequence observed in parasitic plants [41, 42]. Thus, it is advisable that studies of microbial communities in parasitic host plants, and other plant lineages with elevated levels of chloroplast sequence evolution, (e.g. Geraniaceae; [43]), include consideration of potential mismatch between the universal pPNA and host target sequences. In instances where universal pPNA and host sequences differ, designing a modified pPNA complementary to the host sequence is a straightforward and effective way to prevent elevated levels of host contamination. Asteraceae are one of the most species rich families of land plants, including numerous crop species and noxious weeds, and comprise the subjects of several plant microbiome studies [19, 44–46]. Our Asteraceae-specific pPNA, modified to accommodate host variation in organellar DNA sequence without the introduction of bias, thus represents a useful resource for future studies of microbial communities associated with Asteraceae hosts.

## Conclusions

We provide the first empirical test of the efficacy of a universal PNA clamp to block host contamination across a wide range of hosts. We demonstrate that while sequence mismatch between PNA clamp and target plastid DNA reduces deep amplicon sequencing efficiency, clamp function can be recovered through sequence modification without biasing results. We identify six land plant lineages with chloroplast sequence mismatches to universal pPNA, enabling the design of appropriate custom pPNAs for microbiome studies in these taxa. While not without limitations [47], PNA clamps are valuable tools in studies that extract and amplify microbial communities directly from host tissue, and we recommend that future studies include verification that PNA clamp sequences match the target host’s sequence when designing amplicon sequencing experiments.

## Additional files

Additional file 1: **Table S1.** NCBI GenBank accession numbers for the host plant phylogeny. **Table S2.** Rates of plastid and mitochondrial contamination across host plant species. **Table S4.** The effects of host plant species and pPNA type on plastid and mitochondrial contamination. **Table S5.** Results from the analysis of differential abundance of bacterial phyla across pPNA type. **Table S6.** The effects of pPNA type on estimates of α diversity. **Table S7**. The effects of pPNA type on estimates of β diversity. **Figure S1.** PCoA ordination of host plant species with replicate samples amplified with universal and modified pPNA. **Figure S2.** Plastid contamination across plant species is correlated between universal and modified pPNA. (DOCX 257 kb)

Additional file 2: **Table S3.** NCBI GenBank accession numbers for the survey of chloroplast genomes. (XLSX 64 kb)

Additional file 3: R script used for all microbiome bioinformatic and statistical analyses. (R 35 kb)
