# Supplementary material for: Chloroplast sequence variation and the efficacy of peptide nucleic acids for blocking host amplification in plant microbiome studies
Source: Microbiome. 2018 Aug 18;6:144. doi: 10.1186/s40168-018-0534-0 (PMC6098832; doi:10.1186/s40168-018-0534-0)
Supplement: Supplementary file 1 — Table S1. NCBI GenBank accession numbers for the host plant phylogeny. Table S2. Rates of plastid and mitochondrial contamination across host plant species. Table S4. The effects of host plant species and pPNA type on plastid and mitochondrial contamination. Table S5. Results from the analysis of differential abundance of bacterial phyla across pPNA type. Table S6. The effects of pPNA type on estimates of α diversity. Table S7. The effects of pPNA type on estimates of β diversity. Figure S1. PCoA ordination of host plant species with replicate samples amplified with universal and modified pPNA. Figure S2. Plastid contamination across plant species is correlated between universal and modified pPNA. (DOCX 257 kb) [file 40168_2018_534_MOESM1_ESM.docx]

**Additional file 1**

**Supplemental Methods**

**PCR amplification**

We amplified the V4 region of the 16S rRNA gene using a dual-index approach to barcode amplified DNA at the 3’ and 5’ ends. We included mPNA and pPNA clamps to reduce co-amplification of host plant DNA in our reactions. Each 25 μL PCR reaction included the following reagents: 1.5 μL of 10 μM forward indexed primer (515F); 1.5 μL of 10 μM reverse indexed primer (808R); 1 μL of 25 μM mPNA; 1 μL of 25 μM pPNA; 6.5 μL PCR grade H_2_O; 12.5 μL Kappa 2 G Mastermix; 1 μL genomic DNA template. We used the following PCR program: 3 min 95° C; cycle start [15 s 95° C – denaturation; 15 s 78° C - PNA annealing; 15 s 50° C - primer annealing; 15 s 72° C – elongation] cycle end; 5 min 72° C.

We optimized our PCR cycle number to avoid over-amplification of our template DNA, which can obscure differences in relative abundance of taxa and yield chimeric amplicons and PCR artifacts [1]. Based on band intensity on a 1.5% agarose gel, we determined that endosphere samples are optimally amplified using 24 cycles and rhizosphere samples using 20 cycles. We performed all reactions in triplicate using an Eppendorf Mastercycler Pro (Eppendorf, 950030020). We ran each individual reaction on a 1.5% agarose gel at 100 V for 25 mins to check the success of each reaction. On each 96 well amplification plate we also included reactions with a sterile H_2_O sample (negative control), DNA isolated from a pure culture of *Pseudomonas aeuruginosa* (positive control), and DNA isolated from a mock community of known bacteria. These control samples were used to confirm the absence of contamination and to optimize our filtering pipeline. After pooling triplicate reactions, we flourometrically quantified (Invitrogen, PicoGreen, LSP7589) the amplified and pooled product from each individual sample . For each sequencing run, we then added product from all individual samples to a single tube at equal DNA concentration. Pooled libraries were purified with 0.8X AMPure XP beads (Beckman Coulter, A63880), and quantified using the Qubit HS DNA assay (Thermo Fisher Scientific, Q32851). Pooled libraries were sequenced on an Illumina MiSeq using 2 X 150 bp paired-end reads.

**Host Plant Phylogeny**

We downloaded accessions of 3 genes (2 plastid and 1 nuclear) for each of our plant species from NCBI GenBank: ribulose-bisphosphate carboxylase (*rbc*L); maturase K (*mat*K); and internal transcribed spacer (ITS) adjacent to the 5.8S ribosomal RNA gene. For plant species without available sequences we used a closely related congener (Additional file 1: Table S1). We aligned sequences in MEGA v. 6.0 [2] using MUSCLE [3] with default parameters, followed by manually checking alignments. We used BEAST v. 2.1.3 [4] to build a Bayesian phylogenetic tree. For each locus we implemented a standard general time-reversible model (GTR + I + Γ) and an uncorrelated lognormal clock (UCLN) to determine the rate of nucleotide change. We used BEAUTi [4] to constrain the topology and major clade ages of the tree based on a well-resolved plant phylogeny [5]. Our Markov chain Monte Carlo simulation ran for 100 million generations sampled every 10,000 generations, which resulted in 9000 post burn-in trees. We examined stationarity and effective sample sizes of parameter estimates (all ESS > 200) using Tracer v1.6 (http://beast.bio.ed.ac.uk/Tracer). We constructed a consensus tree with mean node heights from the posterior distribution using Tree Annotator v1.6 (Fig. 1).

**References**

1. Polz MF, Cavanaugh CM. Bias in template-to-product ratios in multitemplate PCR. Appl Environ Microbiol. 1998;64:3724–3730.

2. Tamura K, Stecher G, Peterson D, Filipski A, Kumar S. MEGA6: molecular evolutionary genetics analysis version 6.0. Mol Biol Evol. 2013;30:2725–2729.

3. Edgar RC. MUSCLE: multiple sequence alignment with high accuracy and high throughput. Nucleic Acids Res. 2004;32:1792–1797.

4. Drummond AJ, Suchard MA, Xie D, Rambaut A. Bayesian phylogenetics with BEAUti and the BEAST 1.7. Mol Biol Evol. 2012;29:1969–1973.

5. Bell CD, Soltis DE, Soltis PS. The age and diversification of the angiosperms re-revisited. Am J Bot. 2010;97:1296–1303.

**
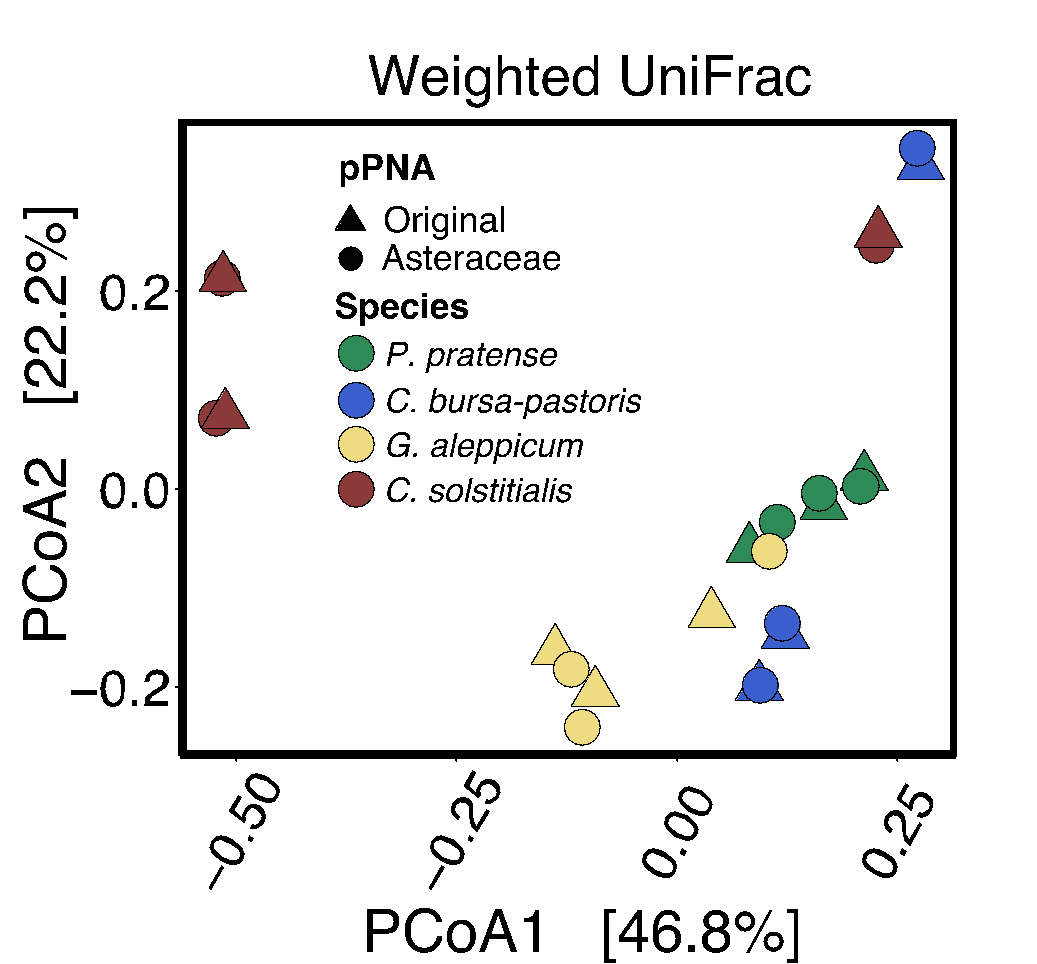
**

**Fig. S1.** pPNA type has no systematic effect on between sample diversity. Each closely situated circle and triangle pair of points represents a single root microbial community sequenced after amplifying with either universal pPNA (triangle) or modified pPNA (circle). Most of the variation among root microbial community composition occurs between host species and individual samples, as opposed to between replicates amplified with either pPNA.

**Fig. S2.** Plastid contamination with Asteraceae-pPNA and universal pPNA are correlated among Asteraceae and non-Asteraceae host plant species.

**Table S1.** NCBI GenBank accessions and collection localities for each host plant species. We queried GenBank for three genetic loci for each host plant species. In cases where data was unavailable, a closely related plant species was used instead. With the exception of *R. virginica* and *C. solstitialis*, all plant tissue used for microbiome analysis came from a roof-top common garden at the University of Toronto Mississauga.

|  | **Genetic locus from NCBI** | | | **Location of tissue collection** | |
| --- | --- | --- | --- | --- | --- |
| **Host plant species** | **rbcL** | **matK** | **ITS** | **Collection site** | **Coordinates** |
| *Amaranthus albus* | JF940785.1 | JF953139.1 | AF210918.1 | UTM | 43.549200, -79.661780 |
| *Asclepias incarnata* | DQ006053.1 | HQ593186.1 | AM396892.1 | UTM | 43.549200, -79.661780 |
| *Asparagus officinalis* | KC704792.1 | JQ276399.1 | HM357931.1 | UTM | 43.549200, -79.661780 |
| *Arctium minus* | HQ589961.1 | HQ593178.1 | KC603906.1 | UTM | 43.549200, -79.661780 |
| *Symphyotrichum ericoides* | EU677041.1 | EU749432.1 | JQ360392.1 | UTM | 43.549200, -79.661780 |
| *Cichorium intybus* | HQ590035.1 | HQ593237.1 | AJ746410.1 | UTM | 43.549200, -79.661780 |
| *Sonchus arvensis* | JX848427.1 | HQ593453.1 | HQ161952.1 | UTM | 43.549200, -79.661780 |
| *Sonchus oleraceus* | KF196024.1 | HQ593454.1 | AJ633306.1 | UTM | 43.549200, -79.661780 |
| *Capsella bursa-pastoris* | HQ589982.1 | HQ593199.1 | FR773706.1 | UTM | 43.549200, -79.661780 |
| *Lepidium densiflorum* | JX848442.1 | HQ593343.1 | NA | UTM | 43.549200, -79.661780 |
| *Sisymbrium officinale* | HQ590273.1 | HQ593442.1 | KF730124.1 | UTM | 43.549200, -79.661780 |
| *Convolvulus arvensis* | HQ590044.1 | HQ593245.1 | AY560274.1 | UTM | 43.549200, -79.661780 |
| *Desmodium canadense* | HQ590061.1 | HQ593266.1 | JF769487.1 | UTM | 43.549200, -79.661780 |
| *Lotus corniculatus* | HQ590168.1 | HQ593352.1 | JN861076.1 | UTM | 43.549200, -79.661780 |
| *Medicago sativa* | HQ590181.1 | HQ593363.1 | AF053142.1 | UTM | 43.549200, -79.661780 |
| *Vicia tetrasperma* | HQ590329.1 | HQ593496.1 | JX506303.1 | UTM | 43.549200, -79.661780 |
| *Oenothera biennis* | HQ590190.1 | HQ593371.1 | EU592030.1 | UTM | 43.549200, -79.661780 |
| *Oenothera perennis* | KM212148.1 | NA | GU176555.1 | UTM | 43.549200, -79.661780 |
| *Plantago major* | HM850266.1 | JN966425.1 | AY101861.1 | UTM | 43.549200, -79.661780 |
| *Plantago rugelii* | DQ006113.1 | EU749330.1 | AY101863 | UTM | 43.549200, -79.661780 |
| *Bromus inermis* | HQ589978.1 | HQ593195.1 | KF713194.1 | UTM | 43.549200, -79.661780 |
| *Festuca arundinacea* | HQ590163.1 | HQ593347.1 | KF713237.1 | UTM | 43.549200, -79.661780 |
| *Phalaris arundinacea* | HQ590203.1 | HQ593381.1 | FJ377669.1 | UTM | 43.549200, -79.661780 |
| *Phleum pratense* | AY395554.1 | HQ593382.1 | EU792341.1 | UTM | 43.549200, -79.661780 |
| *Sporobolus cryptandrus* | HQ590286.1 | HQ593457.1 | GU359208 | UTM | 43.549200, -79.661780 |
| *Persicaria maculosa* | HQ435350.1 | EU749345.1 | EU196892 | UTM | 43.549200, -79.661780 |
| *Geum aleppicum* | HQ590119.1 | HQ593312.1 | NA | UTM | 43.549200, -79.661780 |
| *Geum canadense* | HQ590120.1 | HQ593313.1 | NA | UTM | 43.549200, -79.661780 |
| *Potentilla recta* | HQ590222.1 | HQ593398.1 | FN430784 | UTM | 43.549200, -79.661780 |
| *Solanum dulcamara* | HM850363.1 | HQ593448.1 | AF244742 | UTM | 43.549200, -79.661780 |
| *Rhexia virginica* | U26334.2 | KJ773074.1 | JQ730136.1 | Long Lake, ON Canada | 44.784895, -79.503689 |
| *Centaurea solstitialis* | HM849873.1 | L35880.1 | EU385332.1 | Cazevielle, France | 43.74842, 3.77061 |
|  |  |  |  | Salamanca, Spain | 40.99003, -5.65856 |
|  |  |  |  | Mariposa, CA USA | 37.46178, -119.79218 |

**Table S2.** Plastid contamination rates (proportion of sequenced reads classified as plastid) across host plant species amplified using either universal or Asteraceae-modified pPNA. Note only three non-Asteraceae host plant species were amplified with Asteraceae-modified pPNA.

|  |  | **Universal V4 pPNA** | **Asteraceae V4 pPNA** |
| --- | --- | --- | --- |
| **Family** | **Species** | **Plastid contamination** | |
| Poaceae | *Sporobolus cryptandrus* | 0.03 |  |
| Poaceae | *Phalaris arundinacea* | 0.08 |  |
| Poaceae | *Phleum pratense* | 0.13 | 0.59 |
| Poaceae | *Festuca arundinacea* | 0.03 |  |
| Poaceae | *Bromus inermis* | 0.08 |  |
| Asparagaceae | *Asparagus officinalis* | 0.27 |  |
| Rosaceae | *Potentilla recta* | 0.01 |  |
| Rosaceae | *Geum canadense* | 0.16 |  |
| Rosaceae | *Geum aleppicum* | 0.23 | 0.95 |
| Fabaceae | *Vicia tetrasperma* | 0.07 |  |
| Fabaceae | *Medicago sativa* | 0.02 |  |
| Fabaceae | *Lotus corniculatus* | 0.02 |  |
| Fabaceae | *Desmodium canadense* | 0.14 |  |
| Melastomataceae | *Rhexia virginica* | 0.62 |  |
| Onagraceae | *Oenothera biennis* | 0.15 |  |
| Onagraceae | *Oenothera perennis* | 0.11 |  |
| Brassicaceae | *Sisymbrium officinale* | 0.00 |  |
| Brassicaceae | *Lepidium densiflorum* | 0.13 |  |
| Brassicaceae | *Capsella bursa-pastoris* | 0.07 | 0.54 |
| Solanaceae | *Solanum dulcamara* | 0.55 |  |
| Convululaceae | *Convolvulus arvensis* | 0.06 |  |
| Plantaginaceae | *Plantago major* | 0.15 |  |
| Plantaginaceae | *Plantago rugelii* | 0.18 |  |
| Apocynaceae | *Asclepias incarnata* | 0.05 |  |
| Asteraceae | *Symphyotrichum ericoides* | 0.94 | 0.25 |
| Asteraceae | *Sonchus arvensis* | 0.80 | 0.41 |
| Asteraceae | *Sonchus oleraceus* | 0.92 | 0.42 |
| Asteraceae | *Cichorium intybus* | 0.93 | 0.24 |
| Asteraceae | *Centaurea solstitialis* | 0.27 | 0.02 |
| Asteraceae | *Arctium minus* | 0.02 | 0.03 |
| Polygonaceae | *Persicaria maculosa* | 0.20 |  |
| Amaranthaceae | *Amaranthus albus* | 0.01 |  |

**Table S4.** Linear mixed model results for the effects of host plant species and pPNA type (universal or Asteraceae-modified) on plastid contamination rates. The model terms Species and Species X pPNA were treated as random effects, and pPNA and Usable reads were treated as fixed. Usable reads is the total number of sequencing reads post-filtering for a given sample.

|  | **Plastid Contamination** | | | | | | | | |
| --- | --- | --- | --- | --- | --- | --- | --- | --- | --- |
|  | All plant species, original pPNA | | | Asteraceae, original and modified pPNA | | | Non-asteraceae, original and modified pPNA | | |
|  | *F/X^2^* | *df* | *P* | *F/X^2^* | *df* | *P* | *F/X^2^* | *df* | *P* |
| Usable reads | 6.15 | 257.00 | **0.01** | 15.15 | 23.70 | **<0.001** | 1.00 | 25.23 | 0.33 |
| Species | 108.00 | 1.00 | **<0.001** | 6.59 | 1.00 | **0.01** | 1.53 | 1.00 | 0.20 |
| pPNA | NA | NA | NA | 31.07 | 4.95 | **0.003** | 23.42 | 2.05 | **0.04** |
| Species X pPNA | NA | NA | NA | 0.00 | 1.00 | 1.00 | 0.00 | 1.00 | 1.00 |

**Table S5.** The effects of host plant species and pPNA (universal or Asteraceae-modified) on the abundance of bacterial phyla. Each row displays the significance of host plant species or pPNA on the abundance of a single bacterial phylum, as well as the estimate of differential abundance between universal versus Asteraceae-modified pPNA. Negative values of differential abundance indicate that a bacterial taxon was found at higher abundance in samples amplified with Asteraceae-modified pPNA. With DESeq2, we performed likelihood ratio tests, comparing a full model to a model reduced by either the Species or pPNA term. With ALDEx2, we performed significance tests with generalized linear models for the effect of Species, and with Welch’s t-tests for the effect of pPNA. Differential abundance is estimated as the log_2_-fold change in read count (DESeq2: LFC) or as the relative difference in read count between experimental factors versus within experimental factors (ALDEx2: Effect). Both DESeq2 and ALDEx2 yielded qualitatively similar results for the effects of host plant species and pPNA on the abundance of bacterial phyla. All *P* values were corrected using the False Discovery Rate. Note that results at the level of bacterial phyla are representative of results found at the level of bacterial class, order, family, genus, and ASV (results not shown).

|  | ***P* value: Species** | | ***P* value: pPNA** | | **Differential abundance: pPNA** | |
| --- | --- | --- | --- | --- | --- | --- |
| Phylum | DESeq2 | ALDEx2 | DESeq2 | ALDEx2 | DESeq2: LFC | ALDEx2: Effect |
| Acidobacteria | <0.001 | <0.001 | 0.99 | 0.92 | -0.41 | -0.06 |
| Actinobacteria | 0.02 | <0.001 | 0.99 | 0.92 | 0.53 | 0.07 |
| Armatimonadetes | 0.82 | 0.33 | 0.99 | 0.90 | 0.39 | 0.11 |
| Bacteroidetes | 0.02 | 0.31 | 0.99 | 0.90 | 0.22 | 0.12 |
| Candidatus_Berkelbacteria | 0.99 | 0.33 | 1.00 | 0.91 | 0.47 | 0.03 |
| Chlamydiae | 0.85 | 0.14 | 0.99 | 0.91 | -3.34 | -0.07 |
| Chlorobi | 0.85 | 0.48 | 1.00 | 0.90 | 0.04 | 0.07 |
| Chloroflexi | 0.02 | 0.03 | 0.99 | 0.91 | -0.14 | -0.01 |
| Cyanobacteria | <0.001 | 0.04 | 1.00 | 0.92 | -0.09 | -0.01 |
| Deinococcus-Thermus | NA | 0.30 | NA | 0.91 | -0.14 | -0.03 |
| Elusimicrobia | 0.46 | 0.32 | 0.99 | 0.92 | 0.48 | 0.02 |
| FCPU426 | 0.01 | 0.09 | 1.00 | 0.92 | 0.19 | 0.01 |
| Fibrobacteres | 0.01 | <0.001 | 0.99 | 0.87 | -3.46 | -0.15 |
| Firmicutes | <0.001 | 0.02 | 1.00 | 0.92 | 0.23 | 0.10 |
| Gemmatimonadetes | 0.04 | 0.33 | 0.99 | 0.91 | -0.81 | -0.10 |
| Nitrospirae | 0.99 | 0.34 | 0.99 | 0.89 | -1.48 | -0.09 |
| Parcubacteria | <0.001 | 0.05 | 0.99 | 0.92 | -0.66 | -0.03 |
| Planctomycetes | 0.08 | 0.01 | 0.99 | 0.91 | -0.17 | 0.01 |
| Proteobacteria | <0.001 | <0.001 | 1.00 | 0.90 | -0.05 | -0.06 |
| Saccharibacteria | NA | 0.21 | NA | 0.92 | 1.21 | 0.02 |
| Spirochaetae | <0.001 | <0.001 | 0.99 | 0.90 | 0.17 | 0.08 |
| TM6_(Dependentiae) | 0.99 | 0.35 | 1.00 | 0.90 | 0.74 | 0.09 |
| Verrucomicrobia | <0.001 | <0.001 | 0.99 | 0.90 | 0.09 | 0.08 |

**Table S6.** Linear mixed model results for the effects of host plant species and pPNA type (universal or Asteraceae-modified) on α diversity with either a non-rarefied or rarefied dataset (see Methods). We measured α diversity as Observed species richness, Inverse Simpson’s diversity, and evenness. The model terms Species and Species X pPNA were treated as random effects, and pPNA and Usable reads were treated as fixed. Usable reads is the total number of sequencing reads post-filtering for any given sample.

|  | Total dataset | | | | | | | | |
| --- | --- | --- | --- | --- | --- | --- | --- | --- | --- |
|  | **α Diversity** | | | | | | | | |
|  | Observed richness (S) | | | Inverse Simpson's (D^-1^) | | | Evenness (D^-1^/S) | | |
|  | *F/X^2^* | *df* | *P* | *F/X^2^* | *df* | *P* | *F/X^2^* | *df* | *P* |
| Usable reads | 150.82 | 52.00 | **<0.001** | 0.12 | 56.84 | 0.73 | 53.02 | 57.00 | **<0.001** |
| Species | 9.16 | 1.00 | **0.002** | 5.65 | 1.00 | **0.02** | 1.40 | 1.00 | 0.20 |
| pPNA | 0.60 | 7.56 | 0.46 | 1.29 | 7.58 | 0.29 | 1.01 | 8.34 | 0.34 |
| Species X pPNA | 0.00 | 1.00 | 1.00 | 0.00 | 1.00 | 1.00 | 0.00 | 1.00 | 1.00 |
|  | Rarefied (800 reads) | | | | | | | | |
|  | **α Diversity** | | | | | | | | |
|  | Observed richness (S) | | | Inverse Simpson's (D^-1^) | | | Evenness (D^-1^/S) | | |
|  | *F/X^2^* | *df* | *P* | *F/X^2^* | *df* | *P* | *F/X^2^* | *df* | *P* |
| Species | 1.93 | 1.00 | 0.16 | 6.04 | 1.00 | **0.01** | 3.76 | 1.00 | **0.05** |
| pPNA | 0.50 | 7.52 | 0.50 | 1.35 | 7.75 | 0.28 | 0.58 | 8.25 | 0.47 |
| Species X pPNA | 4.07 | 1.00 | **0.04** | 0.00 | 1.00 | 1.00 | 0.00 | 1.00 | 1.00 |

**Table S7.** Linear mixed model and PERMANOVA results for the effects of host plant species and pPNA type (universal or Asteraceae-modified) on β diversity with either a non-rarefied or rarefied dataset (see Methods). For the linear mixed models, we measured β diversity as a sample’s position along the first three PCoA axes using the Weighted UniFrac distance. The model terms Species and Species X pPNA were treated as random effects, and pPNA and Usable reads were treated as fixed. Usable reads is the total number of sequencing reads post-filtering for any given sample.

|  | Proportional-abundance weighted (common RSVs) | | | | | | | | | | | |
| --- | --- | --- | --- | --- | --- | --- | --- | --- | --- | --- | --- | --- |
|  | **β Diversity** | | | | | | | | | | | |
|  | Weighted UniFrac PCoA 1 | | | Weighted UniFrac PCoA 2 | | | Weighted UniFrac PCoA 3 | | | PERMANOVA | | |
|  | *F/X^2^* | *df* | *P* | *F/X^2^* | *df* | *P* | *F/X^2^* | *df* | *P* | *Pseudo-F* | *R^2^* | *P* |
| Usable reads | 0.86 | 52.22 | 0.36 | 0.00 | 50.51 | 0.95 | 3.17 | 68.53 | 0.08 | 1.50 | 0.01 | 0.15 |
| Species | 0.33 | 1.00 | 0.60 | 13.60 | 1.00 | **<0.001** | 1.92 | 1.00 | 0.20 | 3.46 | 0.27 | **0.001** |
| pPNA | 1.49 | 8.58 | 0.26 | 1.02 | 7.54 | 0.34 | 3.97 | 8.02 | 0.08 | 1.70 | 0.02 | 0.10 |
| Species X pPNA | 0.00 | 1.00 | 1.00 | 0.00 | 1.00 | 1.00 | 0.15 | 1.00 | 0.70 | 0.55 | 0.04 | 0.99 |
|  | Rarefied (800 reads) | | | | | | | | | | | |
|  | **β Diversity** | | | | | | | | | | | |
|  | Weighted UniFrac PCoA 1 | | | Weighted UniFrac PCoA 2 | | | Weighted UniFrac PCoA 3 | | | PERMANOVA | | |
|  | *F/X^2^* | *df* | *P* | *F/X^2^* | *df* | *P* | *F/X^2^* | *df* | *P* | *Pseudo-F* | *R^2^* | *P* |
| Species | 0.49 | 1.00 | 0.50 | 13.50 | 1.00 | **<0.001** | 0.88 | 1.00 | 0.30 | 3.22 | 0.27 | **0.001** |
| pPNA | 1.11 | 8.95 | 0.32 | 1.41 | 7.89 | 0.27 | 3.75 | 8.23 | 0.08 | 1.65 | 0.02 | 0.09 |
| Species X pPNA | 0.00 | 1.00 | 1.00 | 0.00 | 1.00 | 1.00 | 0.38 | 1.00 | 0.50 | 0.63 | 0.05 | 0.99 |
